# Identification of Novel Biomarkers for Priority Serotypes of Shiga Toxin-Producing *Escherichia coli* and the Development of Multiplex PCR for Their Detection

**DOI:** 10.3389/fmicb.2018.01321

**Published:** 2018-06-26

**Authors:** Matthias Kiel, Pierre Sagory-Zalkind, Céline Miganeh, Christoph Stork, Andreas Leimbach, Camilla Sekse, Alexander Mellmann, François Rechenmann, Ulrich Dobrindt

**Affiliations:** ^1^Institute of Hygiene, University of Münster, Münster, Germany; ^2^Genostar Bioinformatics, Montbonnot-Saint-Martin, France; ^3^Norwegian Veterinary Institute, Oslo, Norway

**Keywords:** STEC, O157, non-O157, multiplex PCR, comparative genomics

## Abstract

It would be desirable to have an unambiguous scheme for the typing of Shiga toxin-producing *Escherichia coli* (STEC) isolates to subpopulations. Such a scheme should take the high genomic plasticity of *E. coli* into account and utilize the stratification of STEC into subgroups, based on serotype or phylogeny. Therefore, our goal was to identify specific marker combinations for improved classification of STEC subtypes. We developed and evaluated two bioinformatic pipelines for genomic marker identification from larger sets of bacterial genome sequences. Pipeline A performed all-against-all BLASTp analyses of gene products predicted in STEC genome test sets against a set of control genomes. Pipeline B identified STEC marker genes by comparing the STEC core proteome and the “pan proteome” of a non-STEC control group. Both pipelines defined an overlapping, but not identical set of discriminative markers for different STEC subgroups. Differential marker prediction resulted from differences in genome assembly, ORF finding and inclusion cut-offs in both workflows. Based on the output of the pipelines, we defined new specific markers for STEC serogroups and phylogenetic groups frequently associated with outbreaks and cases of foodborne illnesses. These included STEC serogroups O157, O26, O45, O103, O111, O121, and O145, Shiga toxin-positive enteroaggregative *E. coli* O104:H4, and HUS-associated sequence type (ST)306. We evaluated these STEC marker genes for their presence in whole genome sequence data sets. Based on the identified discriminative markers, we developed a multiplex PCR (mPCR) approach for detection and typing of the targeted STEC. The specificity of the mPCR primer pairs was verified using well-defined clinical STEC isolates as well as isolates from the ECOR, DEC, and HUSEC collections. The application of the STEC mPCR for food analysis was tested with inoculated milk. In summary, we evaluated two different strategies to screen large genome sequence data sets for discriminative markers and implemented novel marker genes found in this genome-wide approach into a DNA-based typing tool for STEC that can be used for the characterization of STEC from clinical and food samples.

## Introduction

Shiga toxin-producing *Escherichia coli* (STEC) have a serious global health impact. An infection with STEC can lead to diarrhea, hemorrhagic colitis and in some cases hemolytic uremic syndrome (HUS) (Croxen et al., 2013). Additionally STEC have the potential to cause large outbreaks with hundreds of hospitalizations and deaths (Terajima et al., 2014; Fruth et al., 2015; Heiman et al., 2015; Yeni et al., 2016). Most of these outbreaks and severe cases of disease worldwide are caused by a limited number of strains including serogroup O157 and the so-called “Big Six” serogroups O26, O45, O103, O111, O121, and O145 (Brooks et al., 2005; Karch et al., 2005). But their prevalence varies among countries and shows geographical clustering (Johnson et al., 2006). Due to the high numbers of infections and hospitalizations caused by STEC O157 and the Big 6 serogroups, these priority serogroups have often been termed clinically relevant STEC serogroups (Lin et al., 2011a; Kerangart et al., 2016). However, many other STEC variants are also pathogenic (Blanco et al., 2004; Johnson et al., 2006; Mellmann et al., 2008).

In order to distinguish between STEC variants associated with severe disease, e.g., HUS, and less virulent STEC, which only cause diarrhea or even non-pathogenic STEC, all STEC isolates from HUS patients in Germany have been systematically collected between 1996 and 2007 and comprehensively analyzed (Mellmann et al., 2008). This resulted in the establishment of the HUS-associated *E. coli* (HUSEC) collection, which comprises 42 reference strains and covers the phylogenetic and genotypic diversity of STEC isolates associated with HUS occurring in Germany (and probably in the other European countries as well) in that period (Mellmann et al., 2008). About three quarters of these isolates represent the STEC priority serotypes mentioned above. But, the HUSEC collection also comprises less frequently isolated variants with the potential to cause severe disease in humans and outbreaks, such as O98:H- or OR:H- STEC isolates of sequence type (ST) 306 (Mellmann et al., 2008; Bai et al., 2018) as well as enteroaggregative *E. coli* (EAEC)-STEC hybrid of serotype O104:H4, which caused a major STEC outbreak in 2011 (Buchholz et al., 2011; Mellmann et al., 2011; Werber et al., 2012). The HUSEC collection, which describes the genotypic and phylogenetic diversity of STEC with the potential to cause outbreaks, was a prerequisite for the rapid and unambiguous identification of the O104:H4 outbreak clone in June 2011 (Bielaszewska et al., 2011). Due to the focus of routine STEC detection on the predominant “Big Five” serotypes at that time, rapid identification of the O104:H4 outbreak strain was severely impaired in many routine diagnostic labs. This example of insufficient STEC identification indicates that reliable hazard characterization requires the determination of discriminatory marker combinations which allow unambiguous discrimination of STEC variants accounting for the majority of outbreaks and severe cases of disease in humans.

As a food borne pathogen, STEC are able to infect humans via various transmission routes, including contaminated meat, vegetables, water, dairy products as well as animal contact (Buchholz et al., 2011; Butcher et al., 2016; Kintz et al., 2017). The severity of STEC-mediated disease does not solely depend on the expression of Shiga toxin (Stx). Several additional virulence factors like intimin (Eae), AaiC and other AggR-dependent factors can also contribute to STEC pathogenesis (Beutin and Martin, 2012). These virulence factors are mostly encoded on mobile genomic elements like bacteriophages, genomic islands or virulence plasmids (Jerse et al., 1990; Schmidt et al., 1995; Ahmed et al., 2008; Karch et al., 2012; Eppinger and Cebula, 2015).

In an outbreak investigation a reliable and quick detection of STEC in food products and food processing environments is needed for source attribution. However, detection of food borne pathogens such as STEC is challenging due to a low infectious dose and a possible heterogeneous distribution in the source material (Harris et al., 2003). Within the European Union (EU), the current detection standard of STEC in food related samples is the ISO TS 13136:2012 which includes a two-step PCR detection of first *stx* and *eae* genes, and samples positive for both genes are subject of detection of O157, O26, O103, O111, and O145 serogroup genes *wzx* or *wzy* in combination with bacterial cultivation (European Committee for Standardization, 2012). Many other detection methods were developed, including conventional multiplex PCRs (Sanchez et al., 2015), real-time PCRs (Lin et al., 2011b), Luminex microbead-based suspension arrays (Lin et al., 2011b; Fratamico et al., 2014) as well as microarray-based approaches (Bugarel et al., 2010; Fratamico and Bagi, 2012; Geue et al., 2014) to increase the speed and reliability. Existing methods can already detect more STEC serogroups than included in the ISO protocol, but most focus on the same marker genes like *stx*, *eae*, *wzx*, or *wzy* (Wang et al., 2013). Furthermore, some PCR-based detection methods, that detect Shiga toxin genes lack a specific *E. coli* amplification control like, e.g., *uidA* (Aranda et al., 2007; Lefterova et al., 2013), which can potentially produce false positive results due to detection of environmental Stx phages (Martinez-Castillo et al., 2013).

Besides detecting selected virulence markers, which are often located on mobile elements, the determination of phylogenetic lineages can support STEC strain characterization. Due to the fact that *E. coli* phylogeny strongly restricts the flexible gene content of individual strains, *E. coli* genome content correlates with the strain’s phylogeny (Touchon et al., 2009; Leopold et al., 2011; Didelot et al., 2012b). Consequently, determination of phylogenetic lineages, i.e., allocation to a sequence type (ST) or clonal complex (CC) based on nucleotide- or genome sequence information (Wirth et al., 2006; Kaas et al., 2012; Clermont et al., 2015) can support STEC typing by correlating the presence of horizontally transferable virulence markers with relevant STEC clones. Thus the combination of virulence, serogroup and phylogenetic markers increases the reliability of strain characterization.

In recent years whole genome sequencing (WGS) has become increasingly popular for characterization of STEC (Didelot et al., 2012a; Hasman et al., 2014; Lambert et al., 2015; Chattaway et al., 2016; Lindsey et al., 2016). WGS methods can reduce the time needed for characterization of STEC and provide data to support subsequent analysis like SNP calling, Multilocus Sequence Typing (MLST) and more, which makes this technology extremely valuable for comprehensive characterization and identification of food borne pathogens, like STEC. But as a major drawback genome sequence-based analyses have to be conducted in an advanced laboratory setting, requiring highly specialized bioinformatics expertise or expensive commercial software (Franz et al., 2014; Eppinger and Cebula, 2015; Parsons et al., 2016; Newell and La Ragione, 2018). Furthermore as long as portable sequencing devices like Oxford nanopore are not commonly used and error-prone (Laver et al., 2015; Lu et al., 2016; de Lannoy et al., 2017), other ubiquitously usable and cheap DNA-based methods need to be developed for an on-site hazard characterization.

The objective of our study was to improve the detection and hazard characterization of STEC by identifying novel global STEC markers in addition to the Shiga toxin gene, *stx*. We designed and compared two bioinformatic pipelines to detect novel discriminative marker gene combinations for STEC in a genome-wide approach. Due to the high genomic plasticity of STEC we could not discover such global STEC marker(s). Consequently, we aimed at the identification of discriminative markers of genotypically more homogenous STEC subgroups represented by the HUSEC collection, and thus performed comparative genomic analyses of isolates allocated to the same O serogroup or ST/CC. It is noteworthy that the NCBI database composition is biased toward major STEC clones and strains, comprising high numbers (*n* > 100) of genome sequences of priority serotypes of STEC incl. O157 and O104, whereas multiple genome sequences of less frequently occurring STEC variants included into the HUSEC collection are scarce. Therefore, we had to restrict our analysis to those subgroups for which multiple genome sequences were publicly available and included 14 STEC subgroups according to their O-antigen or their clonal lineage (ST or CC) into our analysis. This set of STEC variants associated with severe illness and/or outbreaks includes the O157, US priority 6 and O104:H4 serotypes, their corresponding CCs as well as O98:H-/OR:H- (ST306) isolates. We determined specific markers and developed a multiplex PCR (mPCR) for typing isolates of these STEC subgroups. The performance of the mPCR was verified with well-defined clinical isolates and, as a proof of concept, its applicability to food matrices was shown with spiked milk samples.

## Materials and Methods

### Bioinformatics Pipeline A: Collection of Genomes

**FIGURE 1:**
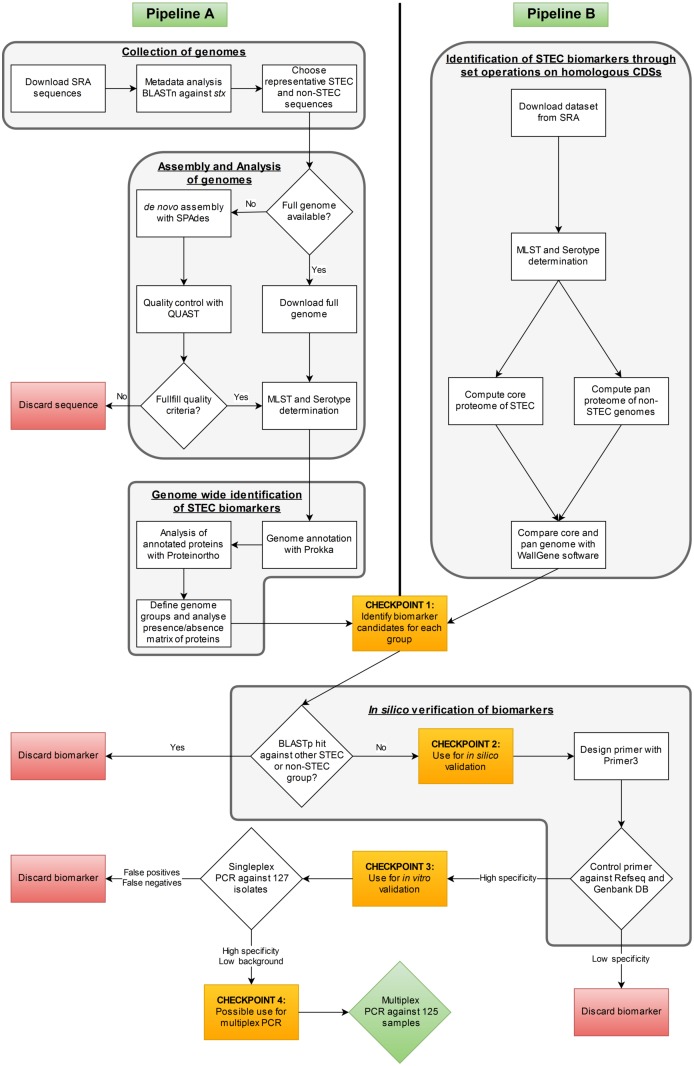
Workflow of the comparative genomic analyses applied in this study for STEC biomarker identification.

After an extensive metadata search and BLASTn analysis with a 95% identity cut-off against Shiga toxin 1 + 2 and intimin-encoding gene alleles of strains Sakai and EDL-933 a subset of 166 STEC genome sequences were chosen from 10,282 *E. coli* genome entries (as of December 2014) available from NCBI’s Sequence Read Archive (SRA) database to represent different STEC O-serogroups (**Figure 1**). Depending on the metadata available, these strains were grouped according to the disease of the patient into HUS-associated STEC or STEC from patients with diarrhea, but not developing HUS. Additionally, a control test set of 82 non-STEC genome sequences containing intestinal pathogenic *E. coli* (IPEC) variants, different extraintestinal pathogenic *E. coli* (ExPEC) isolates and non-pathogenic strains was compiled (**Supplementary Table S1**). The NCBI Reference Sequence database (RefSeq) was used to find and extract complete genomes of the selected *E. coli* strains. If sequences were not available as complete genomes, the sequence reads from the corresponding SRA database entry were *de novo* assembled (see below).

### Bioinformatics Pipeline A: Assembly of Genomes

In a first step SRA raw reads were analyzed with the FastQC software (v0.11.5) (Andrews, 2010) and raw reads with an rejected per base sequence quality were discarded. We compared the assembly results of velvet (v1.2.10) (Zerbino and Birney, 2008) and SPAdes (v3.5) (Bankevich et al., 2012) with and without quality trimming. The results showed that assemblies with SPAdes without previous quality trimming gave fewer and longer contigs than any other combination (data not shown). Therefore, the raw reads were finally assembled with SPAdes (v3.5) with the built-in “–careful” parameter which realigns reads to correct the assembly using the BWA short-read aligner (Li and Durbin, 2009). Due to the fact that gene finding on short contigs can be problematic as the chance to detect multiple ORFs increases with decreasing sequence length and such predicted ORFs are often artifacts we discarded contigs smaller than 1 kb. The quality of the final assemblies was controlled with QUAST v2.3 (Gurevich et al., 2013) (**Figure 1**). *De novo* assembled genomes were considered for analysis if the number of contigs was <1000 with N50 values > 5000 bp and L50 values < 150. The average assembled genome sequence had 173 contigs, an N50 value of 131,691 base pairs (bp) and an L50 value of 19.

### Bioinformatics Pipeline A: Analysis of Genomes

All genomes were analyzed with the SeqSphere+ Software v3.1.0 (Ridom GmbH, Münster, Germany^1^) to allocate the corresponding clonal lineages of the isolates based on MLST. Genomes lacking MLST typing results were discarded. The web-based SerotypeFinder^2^ was used for *in silico* serotyping (Joensen et al., 2015) (**Figure 1**). Virulence gene sequence data were downloaded from the VirulenceFinder database (Joensen et al., 2014) and a BLASTn search was performed to identify the 23 *stx*1, 121 *stx*2 and 45 intimin (*eae*) alleles present in the database in the assembled genomes (**Supplementary Table S1**).

### Bioinformatics Pipeline A: Genome Wide Identification of STEC Biomarkers

Pipeline A consists of three steps (**Figure 1**). First, the 248 genomes were annotated with Prokka v1.11 (Seemann, 2014). The Prokka-generated Genbank file was used to extract the coding sequences (CDS) with cds_extractor v0.7.1^3^ (Leimbach et al., 2017). In the second step, Proteinortho (v5.11) (Lechner et al., 2011) was used to detect orthologous proteins within given genomes via a bidirectional BLASTp analysis. BLASTp parameters of 80% identity and 40% coverage were chosen, which can distinguish *stx1* and *stx2* alleles used in our test set, to determine orthologs. The presence/absence matrix of all orthologs within the strain panel, created by Proteinortho, was used in the last step of the pipeline by a customized Perl script to categorize orthologs according to a maximum of four user-specified genome groups^4^ v0.1. For the analysis, a strict inclusion cut-off of 1 and an exclusion cut-off of 0 were used, meaning that proteins have to be present in all genomes of their own group and absent in all genomes of other groups^5^. In this analysis one to three STEC subgroups were always run against the same subset of 33 non-STEC control strains (**Supplementary Table S1**).

### Bioinformatics Pipeline B: Identification of STEC Biomarkers Through Set Operations on Homologous CDSs

In a first step, the similarity of the annotated genomes of 21 HUS-associated STEC, 22 other clinical STEC isolates and 20 non-STEC isolates (**Supplementary Table S1**) to the genome of reference STEC O157:H7 strain Sakai (NC_002695) was computed using Genostar’s WallGene software. WallGene computes similarity using BLASTp to compare all CDSs of *E. coli* O157:H7 model strain Sakai against all CDSs of the other strains in the dataset. For this analysis, 80% identity and 40% coverage cut-offs were used to assess homology. In a second step, the results of the homology search were used to compute both, the STEC core proteome and the “pan proteome” of the non-STEC control group (**Figure 1**). The core proteome was defined as the set of gene products from the reference strain with orthologs present in at least 80% of the STEC strains. The control pan proteome consists of the set of gene products of the reference strain with at least one ortholog in any member of the non-STEC control group. The third step represented the identification of candidate biomarkers specific for clinically relevant STEC by extracting CDSs present in the STEC core proteome, but absent from the pan proteome of the non-STEC strain set. The second and third steps of the analysis have been performed using an in-house Python script (available on demand).

### *In Silico* Verification of Biomarkers

As a control step after biomarker identification by the two pipelines, the specificity of the biomarker candidates was verified *in silico*^6^. For this purpose the presence of a biomarker candidate protein was investigated by BLASTp analysis. If a BLASTp match displayed >80% identity and >80% coverage in any STEC or non-STEC control genome used in this study the biomarker was discarded. For each of the specific biomarkers, primer pairs were designed with the web-based tool Primer3^7^. The designed primer pairs were then tested *in silico* against all *E. coli* genomes present in the *E. coli* collection of reference strains (ECOR) (72 genomes), in the Diarrheagenic *E. coli* (DEC)-collection (77 genomes) and in the RefSeq database (739 genomes) as well as in the GenBank database (1,951 genomes) (**Supplementary Table S2**) with the EMBOSS primersearch v6.6.0.0 software (primer sequences in **Table 1**) (Rice et al., 2000). Additionally, the final biomarkers have been validated *in silico* by BLASTn analysis against all the genomes of the enterobacterial genera *Shigella*, *Salmonella*, *Proteus*, *Klebsiella*, *Enterobacter*, *Citrobacter*, *Serratia*, and *Yersinia*, which are deposited in the NCBI Nucleotide Collection database (**Supplementary Table S3**).

**Table 1:** PCR primer sequences and concentrations used in this study.

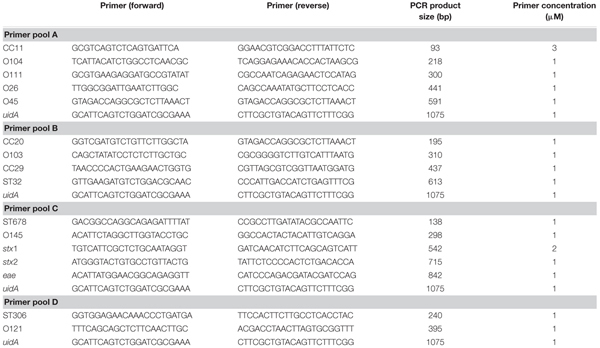

### Primer Design for Shiga Toxin, Intimin and *uidA*

Well-established STEC biomarkers include the Shiga toxin- and intimin-encoding genes. Allelic variants of *stx*1, *stx*2, and *eae*, were downloaded from the VirulenceFinder database (Joensen et al., 2014). 23 *stx*1 allelic variants, the 33 major *stx*2 allelic variants present in our STEC genome test set (**Supplementary Table S4**), and all 45 allelic variants of *eae* were aligned with the command line version of Clustal Omega (clustalo v1.2.1) (Sievers et al., 2011). As an internal amplification control the *E. coli* specific beta-D-glucuronidase-encoding gene *uidA* was used and all 246 *uidA* sequences present in our set of *E. coli* genomes were aligned with Clustal Omega (clustalo v1.2.1). The alignments were used to create a majority rule-based consensus sequence for each gene with the EMBOSS tool consambig (v6.6.0.0) (Rice et al., 2000). For each of these consensus sequences, primers were designed within conserved regions with Primer3^7^. The designed primer pairs were then tested *in silico* as described previously (Rice et al., 2000). Our design of *stx*1- and *stx*2-specific primers considered all ten Stx subtypes defined by Scheutz et al. (2012). Whereas our *stx*1-specific primers allow detection of all *stx*1 alleles, the *stx*2-specific primer pair detects the vast majority of *stx*2 allelic variants except *stx*2f and rare *stx*2d and *stx*2e alleles (for details see **Supplementary Table S4**).

### Phylogenetic Characterization of Representative *E. coli* and STEC Strains

Twenty representative STEC and 31 Shiga toxin-negative *E. coli* strains covering the genomic and phylogenetic diversity of *E. coli* as well as *E. fergusonii* ATCC 35469 and *E. albertii* EC06-170 as outgroups were selected to create a phylogenetic representation of the *stx* distribution. Prokka-generated gff files were used as input for roary v3.11.0 to create a fast core gene alignment with MAFFT using standard parameters (Page et al., 2015). The core genome of these 53 strains consisted of 1193 genes. RaxML v7.2.8 was used with the GTRGAMMA parameter to calculate a bootstrapped majority rule consensus tree with 100 bootstrap replicates (Stamatakis, 2014).

### Singleplex PCR

The singleplex PCRs were performed in a 10 μl volume containing 5 μl GoTaq^®^ G2 Green Master Mix (Promega, Mannheim, Germany), 1 μl forward primer (10 μM), 1 μl reverse primer (10 μM), 2 μl H_2_O and 1 μl DNA sample (20 ng/μl) with the following cycling conditions: initial DNA denaturation at 95°C (180 s), 28 elongation cycles incl. 95°C (30 s), 58°C (30 s), 72°C (time adjusted to product size 1 kb/min), followed by a final elongation step at 72°C (300 s) in a T100^TM^ Thermal Cycler (Bio-Rad, Munich, Germany). Template DNA from 127 clinical isolates (**Supplementary Table S5**) was isolated with the MagAttract HMW DNA kit according to the manufacturer’s recommendations (Qiagen, Hilden, Germany). PCR products were run on a 1.5% agarose gel and stained with 2% ethidium bromide for visualization of PCR products. As a marker 100-bp DNA Ladder (Thermo Fisher Scientific, Dreieich, Germany) was used.

### Sensitivity Testing of DNA Polymerase and Primer Pairs in Multiplex PCR

Three different polymerases [GoTaq^®^ DNA polymerase (Promega, Mannheim, Germany), OneTaq^®^ DNA polymerase (New England Biolabs, Frankfurt/Main, Germany) and Q5^®^ High-Fidelity DNA polymerase (New England Biolabs, Frankfurt/Main, Germany)] were compared to provide the highest possible sensitivity of the PCR reaction. The PCR reactions were prepared according to the manufacturers’ standard protocols. Primer pools and primer concentrations are shown in **Table 1**. For these experiments two different DNA templates were used. First, DNA was extracted according to the standard protocol of the MagAttract HMW DNA Kit (Qiagen, Hilden, Germany) from nine STEC reference strains cultivated overnight in lysogeny broth (LB). These strains cover the clinically most relevant STEC subtypes, which have been included in this study (**Table 2**). The DNA concentrations were measured with a Nanodrop 2000 Spectrophotometer (Thermo Fisher Scientific, Dreieich, Germany) and a 10-fold dilution series ranging from 100 to 0.001 ng/μl was prepared. 2 μl of these DNA samples were used as template in a 20-μl PCR reaction. Additionally, LB overnight cultures of the nine STEC reference strains were adjusted to an OD(600 nm) = 1. One milliliter of this bacterial suspension corresponds to approximately 1 × 10^9^ colony forming units (CFU). A 10-fold dilution series was prepared until samples were diluted 1 × 10^-7^ fold, bacterial cells were pelleted at 7.500 rpm for 10 min, and the pellets were resuspended in 100 μl H_2_O and heated at 90°C for 10 min. 2 μl of these bacterial lysates from the dilution series were used as template in a 20 μl PCR reaction. This corresponds to a template DNA range representative of approximately 180,000 CFU/PCR to 1.8 CFU/PCR. The reactions were then subjected to the following two cycling conditions: initial denaturation of DNA at 95°C (180 s), (cycling condition A) 28 elongation cycles incl. 95°C (30 s), 58°C (30 s), 72°C (time adjusted to product size 1 kb/min); (cycling condition B) 35 elongation cycles 95°C (30 s), 58°C (30 s), 72°C (time adjusted to product size 1 kb/min), followed by a final elongation step at 72°C (300 s) in a T100^TM^ Thermal Cycler (Bio-Rad, Munich, Germany).

**Table 2:** Reference strains used in this study.

Reference strains	MLST	Serotype	*stx*	*eae*
HUSEC003	ST11 (CC11)	O157:H7	2	Positive
HUSEC007	ST17 (CC20)	O103:H2	2	Positive
HUSEC011	ST16 (CC29)	O111:H8	1 + 2	Positive
HUSEC017	ST21 (CC29)	O26:H11	1 + 2	Positive
HUSEC021	ST32 (CC32)	O145:H28	2	Positive
HUSEC031	ST306	OR:H-	1	Positive
HUSEC035	ST655	O121:H19	2	Positive
HUSEC041	ST678	O104:H4	2	Negative
LB408196i1	ST301 (CC165)	O45:H2	2	Positive


### Multiplex PCR

The multiplex PCR was performed in 25-μl reactions containing 5 μl 5x Q5^®^ Master mix (New England Biolabs, Frankfurt/Main, Germany), 0.5 μl peqGOLD dNTP mix (Peqlab, Erlangen, Germany), 1 μl forward primer pool, 1 μl reverse primer pool, 0.25 μl Q5^®^ High-Fidelity DNA polymerase (New England Biolabs, Frankfurt/Main, Germany), 16.25 μl H_2_O and 1 μl DNA sample (20 ng/μl). The reactions were then subjected to the following cycling conditions: initial denaturation of DNA at 98°C (30 s), 28 elongation cycles incl. 98°C (10 s), 58°C (20 s), 72°C (45 s), followed by a final elongation step at 72°C (120 s) in a T100^TM^ Thermal Cycler (Bio-Rad, Munich, Germany). The primer pools and concentrations are shown in **Table 1**.

### Detection of STEC in Spiked Semi-Skimmed Milk Samples

Biosafety and institutional security procedures were applied during cultivation and handling of STEC. LB overnight cultures of nine STEC reference strains were adjusted to an OD(600 nm) = 1. A 5-step 10-fold dilution series was prepared ranging from dilution factor 10^-2^ to 10^-6^. 1 ml of diluted bacterial cells was pelleted at 7,500 rpm for 10 min and the pellets were resuspended in 1 ml of semi-skimmed long life milk and incubated for 30 min at room temperature. Bacterial lysis was performed following the Gram-positive bacteria sample protocol and DNA was then isolated with the protocol for tissues of the QIAamp DNA Blood Mini Kit (Qiagen, Hilden, Germany). DNA was eluted in 100 μl H_2_O and 1 μl of isolated DNA were used in a 10 μl mPCR reaction with 28 PCR cycles as previously described, corresponding to a template DNA range representative of approximately 90,000 CFU/PCR to 9 CFU/PCR.

## Results

### Comparison of the Biomarker Identification Strategies for Clinically Relevant STEC Biomarker Identification Based on Comparative Genome Analysis

**FIGURE 2:**
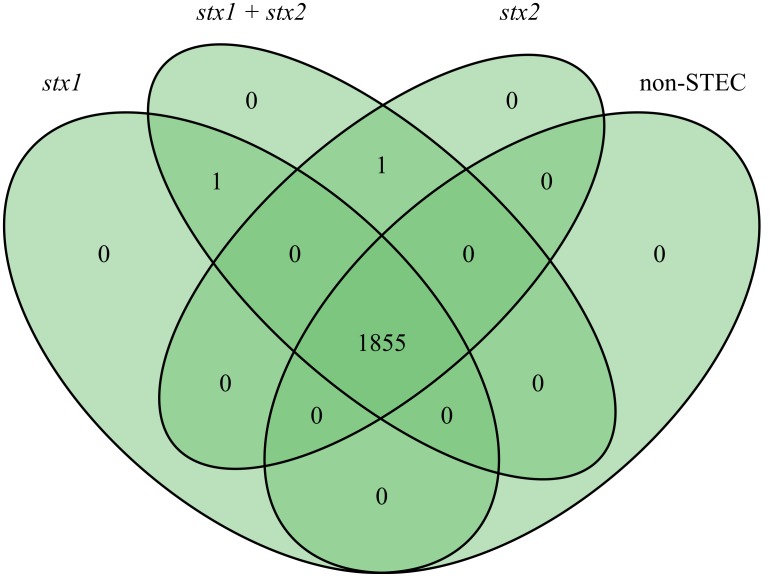
Venn diagram result at checkpoint 1 of bioinformatics pipeline A of 128 genomes grouped into *stx*1-positive STEC (25 genomes), *stx*1+*stx*2 positive STEC (27 genomes), *stx*2 positive STEC (43 genomes) and non-STEC (33 genomes).

**FIGURE 3:**
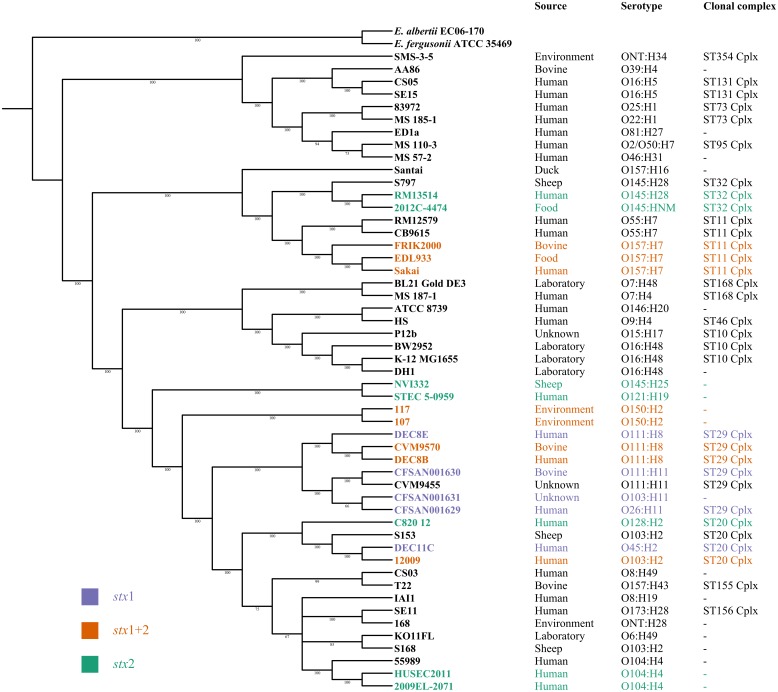
Maximum likelihood tree illustrating the phylogeny of 51 *E. coli* reference strains based on the core genome alignment of 1193 genes. The distribution of STEC strains harboring *stx*1 (purple), *stx*2 (green) or *stx*1 and *stx*2 (orange) is color coded. Additionally, the source, serotype and MLST clonal complex are indicated for all *E. coli* strains.

In order to identify novel global STEC markers in addition to the Shiga toxin genes, we applied two different approaches (pipelines A and B) to independently predict discriminative genes for STEC (**Figure 1**). In a first experiment, 95 STEC genomes were subdivided into three groups according to the presence of the *stx*1 and *stx*2 gene (group 1: only *stx*1-positive; group 2: *stx*1- and *stx*2-positive; group 3: only *stx*2-positive) and tested with pipeline A against 33 non-STEC genomes. Except for Shiga toxin no other gene product could be detected, which was specific for any group (**Figure 2**). Similarly, pipeline B was used to compare the core proteome of 43 STEC strains (*n* = 4,227 homologs) with the pan proteome of 20 non-STEC strains (*n* = 4,887 homologs), using STEC O157:H7 strain Sakai as the reference. Stx-encoding genes were identified as the sole specific STEC biomarkers, thus confirming the results obtained by pipeline A. Similarly, marker genes that distinguish HUS-associated STEC isolates from other clinical and environmental STEC could not be identified by either pipeline (data not shown). Following these results, the distribution of the *stx*1 and *stx*2 genes in representative *E. coli* and STEC strains was determined. Stx-encoding genes were detectable in a phylogenetic diverse group of isolates. Additionally, the presence and combination of *stx* genes was variable even within members of the same serotype or clonal complex (**Figure 3**). Thus, it is not surprising, that except the *stx* alleles themselves no other discriminatory marker could be detected in the diverse group of STEC strains used for the initial analyses. We therefore grouped STEC genomes according to their phylogeny or serogroup in the subsequent experiments.

**FIGURE 4:**
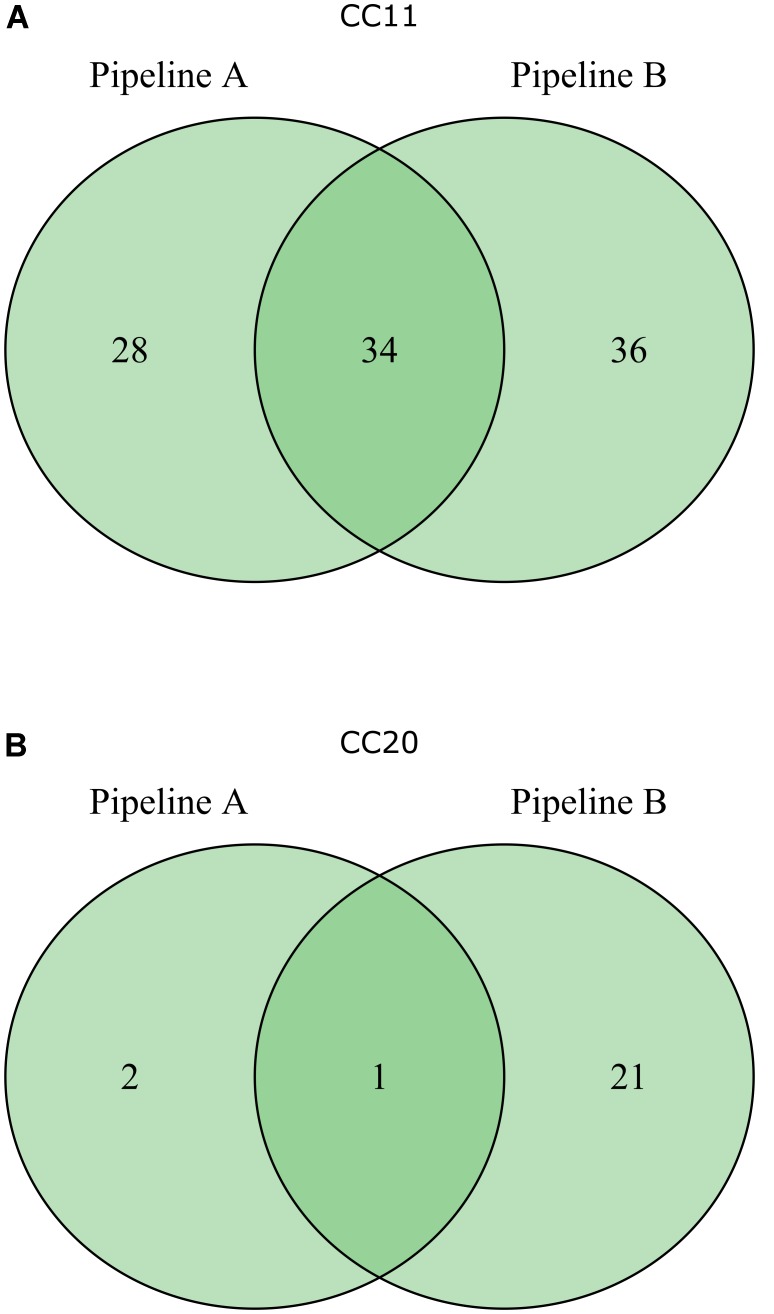
Venn diagrams depicting the results of CC11 **(A)** and CC20 **(B)** marker prediction by pipeline A and pipeline B based on an identical set of 63 *E. coli* genome sequences.

To compare the performance of both pipelines we then analyzed the biomarker prediction for two representative STEC phylogenetic subgroups (CC11 and CC20) with an identical strain set (see panel of 63 genomes used by pipeline B as described above, **Supplementary Table S1**). The CC11 strains were chosen, because they belong to a relatively uniform clonal complex represented by the major clinical O157:H7 strains, whereas the CC20 group were chosen due to their more diverse composition represented by different serogroups, like O103, O128, and O45 (**Figure 3**). Pipeline A detected a lower number of potential STEC markers compared to pipeline B, and only a subset of identical markers was identified by both pipelines (**Figure 4**). One possible explanation could be the use of different assembly and ORF prediction tools in both pipelines. To elucidate this hypothesis, we searched all predicted biomarkers of pipeline B with a BLASTp search in all annotated proteins of pipeline A. Interestingly, we detected differences for 20 putative CC11 and 20 CC20 markers resulting from different assembly and subsequent ORF finding results. Additionally we identified six CC11 and one CC20 biomarker predicted by pipeline B, which were detected in several copies by Prokka and were thus subsequently excluded by Proteinortho in pipeline A. Ten putative CC11 biomarkers exclusively identified by pipeline B were not considered in pipeline A as they were also detected in the control group of non-STEC genomes (**Supplementary Table S6**).

**FIGURE 5:**
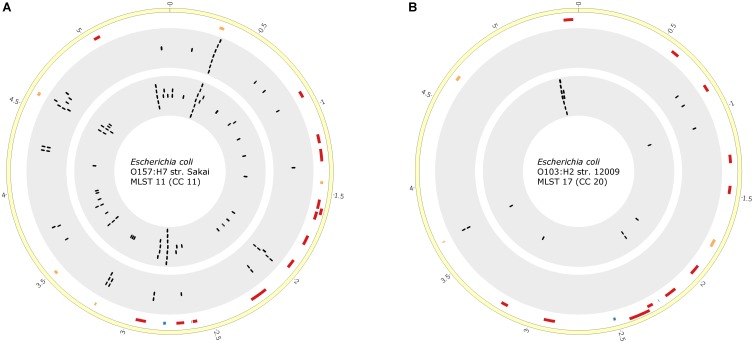
Comparison of marker gene prediction pipeline A based on an enlarged set of genomes with pipeline B. Visualization of specific marker genes identified for STEC isolates of CC11 **(A)** and CC20 **(B)**. The yellow ring represents the chromosome of STEC reference O157:H7 strain Sakai (CC11) **(A)** or O103:H2 strain 12009 (CC20) **(B)**. The second ring depicts bacteriophage-related regions (orange, fragmented; red, intact) within the genomes of the reference strains identified with PHAST (Zhou et al., 2011). Blue markers indicate the localization of O- and H-antigen-related genomic regions. The third ring (gray background) depicts the genomic localization of CC-specific biomarker candidates identified by bioinformatics pipeline A. The fourth ring (gray background) represents the genomic localization of CC-specific biomarker candidates predicted by bioinformatics pipeline B. The plots were generated with CIRCOS (v 0.69) (Krzywinski et al., 2009).

Furthermore, we investigated the impact of different numbers of STEC genomes used for STEC marker prediction with pipeline A. We increased the number of CC11 genomes from 6 to 40 and of CC20 genomes from 4 to 11. The numbers of predicted markers were reduced for CC11 markers (*n* = 42). For CC20, however, two additional markers were identified (*n* = 5). We, again, compared these proteins with the previously identified markers by pipeline B. Several STEC markers were consistently predicted by both approaches (**Supplementary Table S7**). To identify putative marker regions of interests predicted by both pipelines, we analyzed the localization of these marker genes in a closed reference genome. We recognized clusters of marker genes within hotspots (**Figure 5**). Furthermore, many marker genes localized within mobile genomic regions, such as predicted prophages, thus corroborating our finding that the genomic plasticity and phylogeny of *E. coli* has to be considered in the search for discriminatory STEC markers.

As a consequence of the pipeline comparison we decided to continue the comparative genomic analysis with pipeline A. We enlarged our STEC strain set to 248 genomes and classified them into 13 subgroups according to the O-antigen or MLST profile properties, because genome content in *E. coli* markedly correlates with the individual phylogenetic background (Leimbach et al., 2017). These STEC subsets represent the priority serogroups most frequently associated with outbreaks and cases of foodborne illnesses plus their corresponding STs/CCs. A re-analysis of these groups with pipeline A against a subset of 33 non-STEC control strains identified 1,004 biomarker candidates, which could possibly distinguish these priority STEC subgroups. Taken together with the results of pipeline B obtained for CC11 and CC20 1,096 putative discriminatory protein sequences were identified (Checkpoint 1 in **Figure 1** and **Table 3**).

**Table 3:** Amount of biomarkers after checkpoints given in **Figure 1** for each genome subgroup used for bioinformatics pipeline A.

STEC subgroup	Number of genomes	Number of biomarkers after pipeline (Checkpoint 1)	Number of biomarkers after BLASTp verification (Checkpoint 2)	Number of biomarkers used for *in vitro* validation (Checkpoint 3)	Number of biomarkers after *in vitro* validation (Checkpoint 4)
CC11	40	42	9	8	4
CC11 (pipeline B)	6	70	6	0	0
CC20	11	5	0	0	0
CC20 (pipeline B)	4	22	0	4	3
CC29	24	1	1	1	1
ST32	7	155	5	4	2
ST306	9	251	15	5	1
ST678	6	112	4	4	1
O26	10	2	2	2	2
O45	1	307	12	4	3
O103	9	2	2	2	2
O104	8	8	6	6	2
O111	13	8	6	6	3
O121	1	103	14	5	1
O145	11	8	3	3	1
Sum	160	1096	85	54	26


*Pipeline B was only applied for STEC subgroups CC11 and CC20 (as indicated).*

### Selection of the Most Suitable Marker Genes for the Improvement of PCR-Based STEC Typing

Due to the limitation that only three STEC subgroups can be compared with pipeline A at a time, all proteins of an STEC subgroup were used in a custom BLASTp search against all 248 strains excluding the genomes of their specific subgroup^8^. Biomarker candidates were discarded as soon as they had any hit in a different O-antigen or MLST subgroup as targeted (Checkpoint 2 in **Figure 1** and **Table 3**). For all remaining 85 marker gene candidates primer pairs were designed and checked *in silico* against the *E. coli* genome set of the RefSeq and Genbank databases as well as the ECOR and DEC strain collection to validate their specificity (Checkpoint 3 in **Figure 1** and **Table 3**). The 54 biomarker candidates with the highest *in silico* specificity were finally identified for the thirteen STEC subgroups as suitable for *in vitro* verification. These 54 primer pairs were tested in singleplex PCRs against 127 *E. coli* isolates including 42 strains of the HUSEC collection (Mellmann et al., 2008), 83 previously characterized clinical isolates obtained from the German National Consulting Laboratory for HUS-associated *E. coli* as well as the K-12 lab strain MG1655 as a non-pathogenic control and the O104:H4 enteroaggregative *E. coli* (EAEC) strain 55989 to distinguish between *stx*2-positive and *stx*2-negative O104:H4 EAEC (Checkpoint 4 in **Figure 1**, **Table 3** and **Supplementary Table S5**). Based on this *in vitro* primer evaluation, we selected the final primer pair for each STEC subgroup with the highest specificity and best PCR performance (**Figure 1** and **Tables 1**, **4**).

**Table 4:** *In silico* and *in vitro* specificity of the predicted biomarkers.

Primer name	*In silico* validation results obtained/expected (3,087 genomes)	*In silico* specificity	*In vitro* validation results obtained/expected (127 strains)	*In vitro* specificity
CC11	547/556	98.38%	15/15	100.00%
O104	121/121	100.00%	15/15	100.00%
O111	120/120	100.00%	11/11	100.00%
O26	66/66	100.00%	14/14	100.00%
O45	10/10	100.00%	5/5	100.00%
O121	6/5	83.33%	6/6	100.00%
CC20	43/76	56.58%	11/11	100.00%
ST306	9/9	100.00%	11/11	100.00%
O103	30/31	96.77%	11/11	100.00%
CC29	140/136	97.14%	27/27	100.00%
ST32	27/12	44.44%	11/11	100.00%
ST678	119/112	94.12%	13/13	100.00%
O145	26/26	100.00%	18/18	100.00%
*stx*1	389/395	98.48%	48/48	100.00%
*stx*2	585/652	89.72%	87/87	100.00%
*eae*	898/925	97.08%	97/97	100.00%
*uidA*	2898/3015	96.12%	127/127	100.00%


It is noteworthy, that the *in silico* specificity of the primer pairs rarely reached 100%. In four cases it was even below 90% for various reasons. In general, some genomes are poorly annotated in the databases. Additionally, the number of available genome sequences in some groups of reference genomes was quite small (e.g., O121, ST306, and ST32). Furthermore, certain STEC subgroups exhibit a diverse genomic background (e.g., CC20). However, all primer pairs that we selected for our study displayed 100% specificity when tested *in vitro* (**Table 4**).

Additionally, we designed consensus primers for the detection of the typical STEC virulence marker genes *stx*1 and *stx*2 as well as for *eae*. As an internal amplification control, we used primers specific for the *E. coli* beta-D-glucuronidase-encoding gene *uidA*. Most of these primer pairs exhibit an *in silico* specificity of more than 96% tested against the previously described 3,087 genomes, except for the *stx*2 primer pair which cannot detect *stx*2f and some rare allelic variants of *stx*2 (**Supplementary Table S4**).

### Protein Function of Marker Genes and Localization Within Genomes

For each identified biomarker the predicted protein function was determined via BLASTp. The localization of the corresponding genes as well as of the O-antigen cluster within a complete reference genome was identified with a BLASTn search and phage-related regions were detected with PHAST (Zhou et al., 2011). The results are summarized in **Table 5**. Many of the predicted markers are localized in the O-antigen gene cluster. These serogroup-specific marker genes were only identified if STEC genomes were grouped according to the corresponding O-antigen. Whereas the O-antigen polymerase gene is often used for *in silico* serotyping (Wang et al., 2013), we identified other genes mostly involved in O-antigen sugar transfer and biosynthesis (DebRoy et al., 2016) as suitable genomic markers. Genes characteristic for individual sequence types or clonal complexes could be identified within bacteriophage-related or chromosomal regions. These ST- or CC-specific genes mainly encode for hypothetical proteins or a metabolic enzyme (**Table 5**).

**Table 5:** Protein function of biomarker.

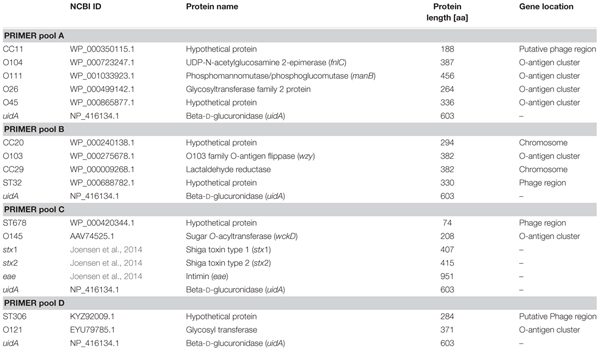

### Validation of Biomarkers in a Multiplex-PCR for Typing of Clinically Most Relevant STEC

In order to reduce the workload for detecting all 13 STEC subgroups as well as the three STEC virulence genes, the primer pairs were combined in four primer pools suitable for mPCR analysis (**Table 1**). To increase the sensitivity of the mPCR three different polymerases GoTaq^®^ DNA polymerase (Promega), OneTaq^®^ DNA polymerase (New England Biolabs) and Q5^®^ High-Fidelity DNA polymerase (New England Biolabs) were examined in two different sensitivity experiments. First, DNA template dilutions ranging from 100 to 0.001 ng were tested in combination with the different DNA polymerases. The Q5 polymerase showed the highest sensitivity of all three polymerases and enabled biomarker detection with as few as 0.1 ng template DNA. In contrast, the OneTaq and GoTaq polymerases required 10–100 ng DNA as template to successfully detect most biomarkers (data not shown). In a second experiment different ranges of CFU per PCR reaction were tested from 180,000 CFU/PCR to 1.8 CFU/PCR. The results of the CFU dilution series corresponded with the DNA dilution series. The Q5^®^ High-Fidelity DNA polymerase showed the highest sensitivity with a reliable detection limit of about 18 CFU/PCR, when 35 PCR cycles were used. Interestingly, the GoTaq^®^ DNA polymerase failed to detect most marker genes even when high CFU concentrations have been used as template. The OneTaq^®^ DNA polymerase was only able to detect the majority of the biomarkers when a high template concentration (corresponding to 180,000 CFU) was used (data not shown).

**FIGURE 6:**
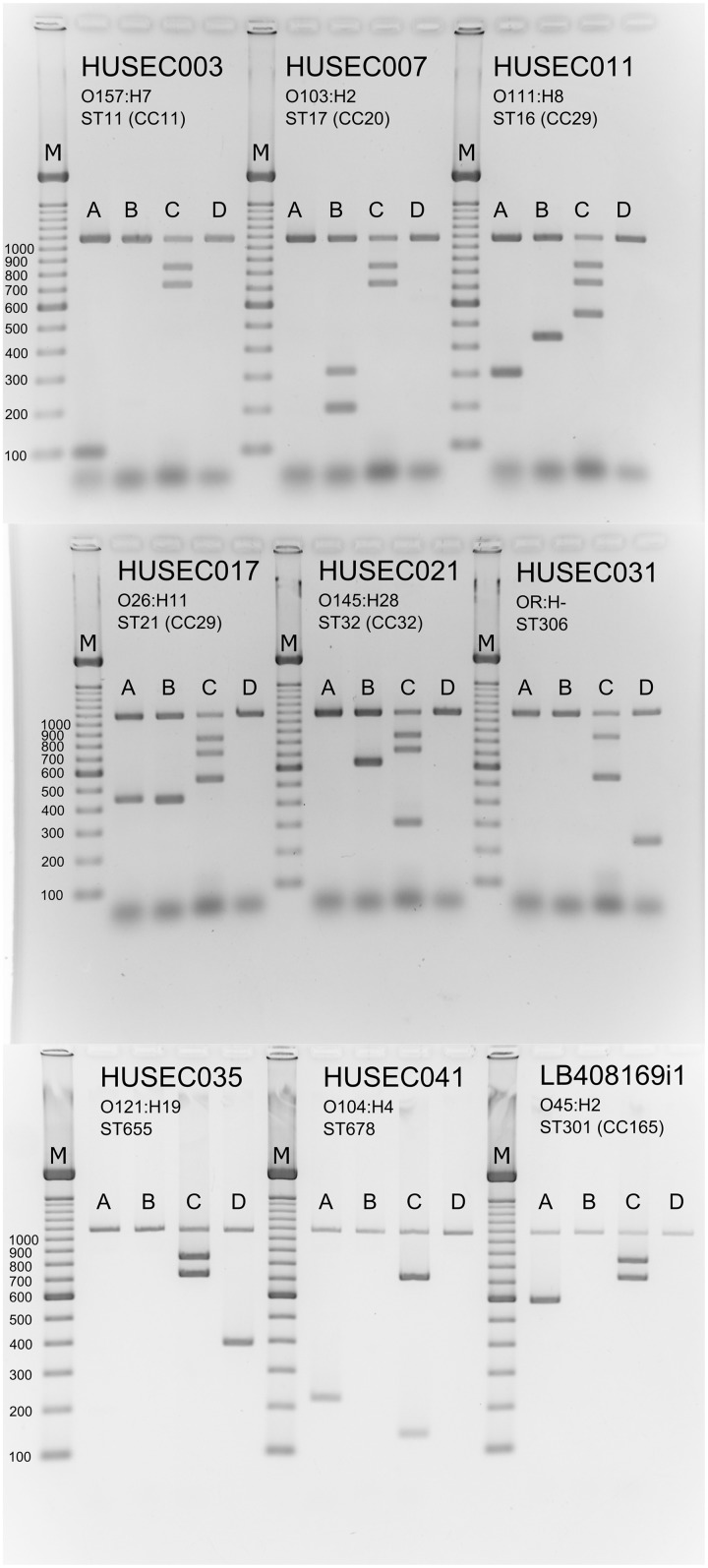
Multiplex PCR pattern of clinically relevant STEC variants. Seventeen primer pairs were designed for the specific detection of the O157, the non-O157 “Big Six” and O104:H4 serogroups or their associated clonal lineages as well as ST306 STEC isolates. All of the primer pairs yield specific gene products indicating the appropriate serogroup or sequence type and generate no unspecific products as visualized by agarose gel electrophoresis. Lane M: 100-bp ladder (Fermentas). Representative STEC reference strains were tested with the four primer pools A–D: HUSEC003 (O157:H7, ST11 (CC11), *uidA* positive, *eae* positive, *stx*2 positive, CC11 positive), HUSEC007 [O103:H2, ST17 (CC20), *uidA* positive, *eae* positive, *stx*2 positive, O103 positive, CC20 positive], HUSEC011 [O111:H8, ST16 (CC29), *uidA* positive, *eae* positive, *stx*1 positive, *stx*2 positive, CC29 positive, O111 positive], HUSEC017 [O26:H11, ST21 (CC29), *uidA* positive, *eae* positive, *stx*1 positive, *stx*2 positive, CC29 positive, O26 positive], HUSEC021 [O145:H28, ST32 (CC32), *uidA* positive, *eae* positive, *stx*2 positive, ST32 positive, O145 positive], HUSEC031 (OR:H-, ST306, *uidA* positive, *eae* positive, *stx*1 positive, ST306 positive), HUSEC035 (O121:H19, ST655, *uidA* positive, *eae* positive, *stx*2 positive, O121 positive), HUSEC041 (O104:H4, ST678, *uidA* positive, *eae* positive, O104 positive, ST678 positive), and LB408169i [O45:H2, ST301 (CC165), *uidA* positive, *eae* positive, *stx*2 positive, O45 positive].

Based on these results, the robustness of the four mPCR primer pools was tested using the Q5^®^ High-Fidelity DNA polymerase with the 127 well characterized clinical STEC *E. coli* isolates that had been used for specificity testing of the individual biomarker primer pairs (**Supplementary Table S5**). A representation of the mPCR results obtained from nine selected reference strains, which cover all 13 defined STEC subgroups and associated sequence types, is shown in **Figure 6**.

### Application of Improved STEC Biomarker Detection to Food

Improved on-site detection of the clinically most prevailing STEC subtypes may facilitate screening of food samples. As a proof-of-concept experiment and to evaluate the effect of a food-related matrix, we tested the detection of STEC marker genes in semi-skimmed milk samples spiked with defined numbers of bacterial cells of nine STEC reference strains ranging from 9 × 10^6^ CFU/ml to 9 × 10^2^ CFU/ml. The mPCR reliably detected all STEC marker genes down to a template concentration of 9,000 CFU/reaction with 28 PCR cycles (**Table 6**). It is noteworthy; that the detection limit was significantly higher compared to pure culture dilution series. However, the sensitivity could be increased to 900 CFU/reaction if the mPCR was run with 35 PCR cycles, but this also led to an increased background (data not shown).

**Table 6:** Results of milk dilution series with 28 PCR cycles.

Marker	PCR product (bp)	90000 CFU/PCR	9000 CFU/PCR	900 CFU/PCR	90 CFU/PCR	9 CFU/PCR
CC11	93	xx	x	-	-	–
O104	218	xx	xx	-	-	–
O111	300	xx	xx	x	-	–
O26	441	xx	xx	(x)	-	–
O45	591	xx	xx	-	–	–
CC20	195	xx	xx	-	–	–
O103	310	xx	xx	-	–	–
CC29	437	xx	xx	x	(x)	–
ST32	613	xx	xx	(x)	–	–
ST678	138	xx	x	-	–	–
O145	298	xx	x	-	–	–
*stx*1	542	xx	xx	x	–	–
*stx*2	715	xx	x	(x)	–	–
*eae*	842	xx	xx	(x)	–	–
ST306	240	xx	xx	x	–	–
O121	395	xx	xx	x	–	–
*uidA*	1075	xx	xx	x	–	–


*xx, strong signal; x, medium signal; (x), weak signal; –, not detectable.*

## Discussion

Detection and typing of STEC by molecular methods is, despite recent advances, still challenging. As intestinal pathogens, they may represent only a minor fraction of the complex and large microbial consortium found in clinical samples of diarrheagenic patients. As food borne pathogens, they have low infectious doses, while at the same time they may be heterogeneously distributed in food samples (Harris et al., 2003). Proper sampling is therefore critical to obtain sufficiently low detection and quantification limits. To date STEC detection in food and clinical samples often includes a time consuming enrichment step. Due to the high genomic plasticity of *E. coli* in general and the frequent presence of multiple *E. coli* strains in one clinical stool or food sample, several currently existing STEC subtyping methods may lead to misinterpretation of results and misidentification of putative outbreak strains, as it was the case with the O104:H4 hybrid outbreak strain in 2011 (Buchholz et al., 2011; EFSA Panel on Biological Hazards, 2013). This furthunderlines that a reliable hazard characterization requires the determination of marker combinations, which allow unambiguous discrimination of STEC variants with the potential to cause disease in humans. According to the recommendation of the European Centre for Disease Prevention and Control (ECDC) the currently used approaches include detection of only a few STEC virulence markers, incl. *stx* and *eae* as well as some serogroup-specific genes (DebRoy et al., 2011; Luedtke et al., 2014; Sanchez et al., 2015; European Food Safety Authority, 2016). These approaches, however, neither allow unambiguous identification of all clinically relevant STEC variants nor their distinction from STEC strains, which are probably non-pathogenic to humans. Nonetheless, the majority of PCR-based detection methods for STEC still focus on *wzy*/*wzx* O-antigen genes (Wang et al., 2013). Whole genome sequence-based strain typing is the state-of-the-art for comprehensive STEC typing in clinical microbiology and also becomes a valuable tool for well-equipped laboratories in food microbiology (Franz et al., 2014; Joensen et al., 2014; Eppinger and Cebula, 2015; Chattaway et al., 2016; Lindsey et al., 2016; Lee et al., 2017; Sekse et al., 2017). Delannoy et al. (2013a,b, 2016b) have already demonstrated that individual combinations of an extended set of known markers beyond the classical STEC marker genes allow the identification of STEC serotypes (O157:H7, O26:H11, O45:H2, O103:H2, O111:H8, O121:H19, O145:H28, and their non-motile derivatives), which are most frequently implicated in outbreaks and sporadic cases of hemorrhagic colitis and hemolytic uremic syndrome worldwide. Additionally they showed that clustered regularly interspaced short palindromic repeat (CRISPR) regions can be used to discriminate the same serotypes as well as O104:H4 (Delannoy et al., 2012a,b, 2016a). Furthermore, Wong et al. (2014) identified a novel O157:H7-specific marker in a genome wide insertion/deletion-based approach. Searching for allelic variation that may support sequence-based typing of different STEC serogroups, Gilmour et al. (2007) reported that also other genes outside the O-antigen cluster (*mdh*, *gnd*, *gcl*, *ppk*, *metA*, *ftsZ*, *relA*, and *metG*) can be used to distinguish different STEC serogroups. Taken together, the growing genomic sequence data offers additive information that may support the identification of discriminative markers for so far less well-described STEC serotypes (Franz et al., 2014).

### In Search for Novel Discriminative Markers for Priority STEC Subgroups

To extend STEC diagnostics in the post-genomic era beyond the detection of the O157:H7 and the “Big Six” non-O157 serogroups, it was thus our idea to take advantage of existing whole genome sequence information and develop a suitable pipeline to integrate high throughput sequence (HTS) data into pathogen detection in combination with strain typing. Accessibility of sufficient and valid genome sequence data for researchers is sometimes limited. At the beginning of our project (December 2014) 10,282 complete and draft *E. coli* genome sequence data sets were publicly accessible. Unfortunately, the majority of these genome sequences lacked sufficient sequence quality and/or metadata availability and thus had to be excluded from our analysis. Furthermore, the vast majority of database entries represented redundant sequence information of some major STEC serogroups (O157, O26, O111, O145, and O104), whereas only sparse or even single database entries with good sequence quality existed for most minor, but also some major STEC variants (e.g., O121 and O45) associated with severe disease in humans. Finally, only a relatively small number of WGS data sets (*n* = 248) was used for our genome comparison. This highlights the limitations of some of the SRA entries and emphasizes the need for regular updates, sufficient sequence quality, and availability of metadata (e.g., disease type, source of isolate). To initially distinguish between (i) STEC and non-STEC, (ii) different STEC clones or (iii) genomes carrying different *stx* alleles, we had to download all the *E. coli* genome entries and manually detect *stx* variants, sero- and/or sequence types. Recently, the search for genomes of interest in the SRA has been facilitated by the Bitsliced Genomic Signature Index (BIGSI) (Bradley et al., 2018). However, an STEC specific database would be advantageous to remedy these problems (Franz et al., 2014). In part this problem is tackled by the GenomeTrakr Database, which aims to collect genomes of four food-borne pathogens (*Salmonella, Listeria, E. coli*/*Shigella*, and *Campylobacter*) together with detailed metadata (Stevens et al., 2017).

The classification of the downloaded genome sequences was the first step toward a systematic and unbiased screening of whole genome sequence data for discriminatory STEC marker genes. We not only considered STEC genome plasticity by including multiple genomes of representatives of the different STEC subgroups and phylogenetic lineages for an unbiased definition of STEC markers. Additionally, we used two pipelines to analyze the HTS data. Pipeline A performed a protein-by-protein bidirectional comparison, whereas pipeline B defined the STEC core proteome and compared this against the non-STEC pan proteome. Different outcomes of both pipelines depend mainly on two factors: First, pipeline A is based on ORF finding by Prokka, which may differ from the ORF definition of the NCBI annotated Refseq and Genbank entries used in pipeline B. Second, subgroup-specific analysis in pipeline A includes an all-against-all comparison of all proteins found in up to four subgroups compared, whereas pipeline B requires definition of an STEC core proteome based on a reference strain prior to comparison with the pan proteome of another group, here all non-STEC strains. Because of this, pipeline A may be more suitable for the identification of markers in rare STEC variants with a less congruent genome content relative to the reference strain. On the other hand, pipeline A will be more computational demanding than pipeline B to detect specific genes for multiple STEC subgroups. Furthermore, pipeline A used a more stringent cut-off (100%) for presence of a marker in the STEC group, whereas pipeline B was run with an 80% cut-off parameter.

Generally, both comparative approaches (pipelines A and B) led to the identification of overlapping, but not completely identical groups of marker genes (**Figures 4**, **5**). The observed outcome mirrors deviating ORF finding results between Prokka and WallGene due to different settings in both tools as well as the different used cut-offs (**Supplementary Table S6**).

To the best of our knowledge similar approaches which translate *in silico* genome comparison data to *in vitro* diagnostics were only rarely done in *E. coli*. Wang et al. (2011) predicted two novel markers for O157:H7 by a comparative BLAST analysis of three O157:H7 genomes against 750 prokaryote genomes. Interestingly, these markers are located in a similar region as the CC11 marker identified in our study (Z0344/Z0372 vs. Z0331). Whiteside et al. (2016) introduced the online platform SuperPhy, which is the first attempt to combine the immense genomic information of *E. coli* with phenotypic traits. In subsequent work they showed the usability of SuperPhy to identify predictive biomarkers for subgroups of *Salmonella enterica* (Laing et al., 2017). Pielaat et al. (2015) did a first step to combine *in vitro* adherence data with genomic SNP data for an improved food safety risk assessment of STEC O157:H7 strains. Furthermore, joint efforts are made within the Global Microbial Identifier (GMI) consortium to progress with the goal to combine NGS, bioinformatics and open data access with standardized food safety (Taboada et al., 2017). In STEC detection the majority of methods concentrate on known virulence factors, whereas our comparative analysis did not *a priori* focus on STEC virulence-related determinants. As the gene content of the *E. coli* flexible genome is dominated by the phylogenetic background (Touchon et al., 2009) and STEC represent a phylogenetically diverse group of pathogens, it was not too surprising that general STEC biomarkers other than *stx* could not be identified for all STEC variants (**Figure 2**). Additionally, HUS-associated STEC could not be distinguished from other clinical STEC strains further supporting previous findings that no virulence factor pattern could be identified to distinguish all STEC responsible for the majority of outbreaks and severe human infections from other STEC with lower potential to cause severe disease in humans (Franz et al., 2015). Consequently, we tried to take advantage of the huge and continuously growing genome sequence data set and decided to search for marker genes characteristic for subgroups of clinically relevant STEC serogroups and/or their corresponding clonal lineages. The lack of publicly available high-quality genome sequence information of multiple independent isolates of the less frequently occurring STEC variants limited our analysis. For those 24 different serotypes of clinically relevant STEC variants represented by the HUSEC collection (Mellmann et al., 2008), we could only run our pipeline with the top seven STEC serotypes. With exponentially increasing WGS data these gaps will likely be closed soon and our pipeline can be applied to detect novel biomarkers for the so far underrepresented STEC types. Until then, the comparative genomic analysis led to the compilation of 54 candidate STEC marker genes specific for the tested subgroups sorted according to sero- or sequence types (**Table 3**).

From the pool of STEC marker candidates identified by pipeline A and B, we *in silico* selected the most suitable marker genes for the relevant serogroups and clonal complexes and confirmed their specificity by PCR using 127 well-characterized clinical STEC isolates. All biomarker primer pairs displayed 100% specificity in our mPCR experiments (**Table 4**). However, the selected CC20 biomarker is not ideally suited for unambiguous STEC typing, because CC20 contains many highly diverse strains of different O serogroups incl. O103, O45, O128, and O145. A marker gene specific for all CC20 strains could not be verified by pipeline A including a larger and more diverse set of genomes (**Table 3**). The comparison of the CC20 core proteome against the non-CC20 pan proteome defined candidate markers, but was based on the genome sequences of the *E. coli* strains PMK-5 (O103:H2), 12009 (103:H2), DEC11C (O45:H2), and STEC_H.1.8 (O128:H8), which represent only a fraction of serotypes included in CC20. Accordingly, the candidate CC20 markers are not conserved in all CC20 isolates and display the lowest *in silico* specificity of all biomarkers (**Table 4**). This observation confirms that the outcome of comparative genomic approaches depends on (i) the number of genomes included into the comparison and (ii) the genomic diversity of the isolates comprised in the different subgroups used.

Interestingly, another typical classification factor for STEC isolates, the source of the isolate, did not influence our analysis. The *in silico* analysis of ST306 biomarkers was solely based on plant-associated and environmental STEC isolates (**Supplementary Table S1**), but the identified biomarker showed 100% specificity for human clinical samples (**Table 4**). Additionally, in our study, STEC strains isolated from bovine, sheep or food samples were present in most STEC subgroups tested (**Supplementary Table S1**) and showed no difference in the presence of the biomarker genes compared to human clinical isolates. This corroborates the universal usability of our described biomarkers to analyze clinical samples as well as food samples.

### Development of a Multiplex PCR for Rapid Typing of Clinically Relevant STEC

Based on our *in silico* analysis of large genome sets available from publicly databases and the PCR-based screening of a large number of well-characterized clinical STEC isolates, we have identified marker combinations, which allow a reliable differentiation of the priority STEC variants described above (**Table 5**). The comparative genomic analysis of a larger panel of genomes also enabled us to improve the specificity and performance of published *uidA*-specific primer pairs (data not shown).

To verify our set of marker genes, we developed a multiplex screening PCR to identify different STEC strains (**Figure 6**). We tested the analytical sensitivity of the four primer pools with pure cultures or purified genomic DNA of clinical isolates and three different DNA polymerases. Depending on the DNA polymerase, number of cycles used for amplification, and the primer pool, the detection limit for reproducible amplification of the markers was as low as 0.01 ng DNA or 18 CFU when the Q5 DNA polymerase and 35 cycles were used (data not shown). As a proof of principle, we further showed the usability of our mPCR to reliably detect STEC marker genes in contaminated milk samples down to 900–9,000 CFUs per PCR, depending on the cycle number and primer pair used (**Table 6**). In a recent mPCR assay for the identification of different mastitis pathogens, the detection limit for *E. coli* from pure cultures was 0.01 ng DNA (Ashraf et al., 2017). The analytical sensitivity of other mPCR-based detection of *E. coli* from spiked milk samples ranges from 10^2^ CFU/ml (Cressier and Bissonnette, 2011; Ashraf et al., 2017) to 10 CFU/ml (Shome et al., 2011). In these studies different DNA extraction and PCR protocols have been used, which can markedly affect the outcome of the assay. Our mPCR results confirm the functionality of the *in silico* predicted biomarkers.

## Conclusion

Our genome-wide search for discriminative STEC markers identified new targets for detection and typing of different STEC subgroups. The combination of these novel chromosomal regions specific for the serogroup and for corresponding clonal groups with the STEC standard markers *stx* and *eae* resulted in a robust, specific and reliable typing of the clinically most relevant STEC variants and can improve risk analysis of STEC isolates by *in silico* typing based on NGS data or by mPCR. Correct and timely identification of STEC isolates is crucial for food microbiology for market access testing as well as for surveillance of STEC-mediated disease. Our primer set and also our mPCR can help to reduce the risk of false positive STEC detection due to free *stx*-converting bacteriophages or *stx*-positive non-*E. coli* members of the *Enterobacteriaceae*. The detection of the *E. coli*/*Shigella*-specific *uidA* marker will indicate whether these species are present or not. As long as DNA sequence-based diagnostics of mixed populations cannot resolve whether relevant markers are present in the same genome, some risk of generating false-positive results, however, will remain. But the combination of virulence- and phylogenetic lineage-related markers of our mPCR scheme supports correct hazard characterization. Our approach offers a greater variety of detectable STEC markers for risk assessment and strain typing by well-equipped and trained laboratories, e.g., in outbreak situations when the outbreak strain has to be identified/detected. Based on the availability of additional genome sequences in the future, the marker gene set can be further extended to STEC subgroups that had to be excluded so far. Whole genome sequencing is becoming the state-of-the-art technology for typing of microbial isolates cultivatable as a pure culture. In routine food microbiology, however, where often more complex samples and different food matrices have to be analyzed, use of whole genome sequence-based typing is still under development. Advanced bioinformatic analyses of HTS data sets retrieved from composite bacterial cultures have to be established to enable meaningful genome analysis and bacterial typing of mixed cultures. Until then, multiplexed DNA-based approaches offer advantages for monitoring throughout food production chains in terms of practicability and on-site usage and costs. Thus, future work will have to focus on the use of the identified biomarkers with on-site detection methods, like LAMP-assays in combination with lab-on-a-chip-based as well as with nanofluidics-based screening technologies to improve and facilitate the detection of STEC in food.

## Author Contributions

MK, CM, CSt, CSe, FR, and UD conceived and designed the experiments. MK, CSt, AM, and UD collection and analysis of samples. MK, PS-Z, and CM performed the experiments. MK, PS-Z, CM, CSt, AL, and UD analyzed the data. MK and UD draft the manuscript. All authors critically revised and approved the final version of the manuscript.

## Conflict of Interest Statement

The authors declare that the research was conducted in the absence of any commercial or financial relationships that could be construed as a potential conflict of interest.

## Footnotes

1 http://www.ridom.de/seqsphere/

2 https://cge.cbs.dtu.dk/services/SerotypeFinder/

3 https://github.com/aleimba/bac-genomics-scripts

4 https://github.com/aleimba/bac-genomics-scripts/tree/master/po2group_stats

5 https://github.com/dobrindtlab/shell_scripts/blob/master/Post-Prokka-Biomarker-Blast.sh

6 https://github.com/dobrindtlab/shell_scripts

7 http://bioinfo.ut.ee/primer3/

8 https://github.com/dobrindtlab/shell_scripts/blob/master/Post-Prokka-Biomarker-Blast.sh

## Supplementary Material

TABLE S1 *In silico* genomes for tests with pipelines A+B.

TABLE S2 *In silico* genomes used from Refseq + GenBank.

TABLE S3 Results primer BlastN against enterobacterial genomes.

TABLE S4 *stx* primer specificity test.

TABLE S5 Clinical isolates tested.

TABLE S6 Comparison pipeline A+B.

TABLE S7 CC11 and CC20 markers detected by pipeline A+B.
